# Gene expression changes associated with the evolutionary loss of a metabolic trait: lack of lipogenesis in parasitoids

**DOI:** 10.1186/s12864-019-5673-6

**Published:** 2019-04-23

**Authors:** Mark Lammers, Ken Kraaijeveld, Janine Mariën, Jacintha Ellers

**Affiliations:** Department of Ecological Sciences, Section Animal Ecology, Vrije Universiteit Amsterdam, De Boelelaan 1085, 1081 HV Amsterdam, The Netherlands

**Keywords:** Fatty acid auxotrophy, Trait loss, Lipogenesis, Parasitoid lifestyle, Pleiotropy, RNA-seq, Comparative transcriptomics

## Abstract

**Background:**

Trait loss is a pervasive phenomenon in evolution, yet the underlying molecular causes have been identified in only a handful of cases. Most of these cases involve loss-of-function mutations in one or more trait-specific genes. However, in parasitoid insects the evolutionary loss of a metabolic trait is not associated with gene decay. Parasitoids have lost the ability to convert dietary sugars into fatty acids. Earlier research suggests that lack of lipogenesis in the parasitoid wasp *Nasonia vitripennis* is caused by changes in gene regulation.

**Results:**

We compared transcriptomic responses to sugar-feeding in the non-lipogenic parasitoid species *Nasonia vitripennis* and the lipogenic *Drosophila melanogaster*. Both species adjusted their metabolism within 4 hours after sugar-feeding, but there were sharp differences between the expression profiles of the two species, especially in the carbohydrate and lipid metabolic pathways. Several genes coding for key enzymes in acetyl-CoA metabolism, such as *malonyl-CoA decarboxylase* (*mcd*) and *HMG-CoA synthase* (*hmgs*) differed in expression between the two species. Their combined action likely blocks lipogenesis in the parasitoid species. Network-based analysis showed connectivity of genes to be negatively correlated to the fold change of gene expression. Furthermore, genes involved in the fatty acid metabolic pathway were more connected than the set of genes of all metabolic pathways combined.

**Conclusion:**

High connectivity of lipogenesis genes is indicative of pleiotropic effects and could explain the absence of gene degradation. We conclude that modification of expression levels of only a few little-connected genes, such as *mcd*, is sufficient to enable complete loss of lipogenesis in *N. vitripennis*.

**Electronic supplementary material:**

The online version of this article (10.1186/s12864-019-5673-6) contains supplementary material, which is available to authorized users.

## Background

In recent years, there has been a growing awareness of the adaptive role of trait loss in evolution [1–4]. Trait loss has been shown to affect resource use efficiency [5], speciation [6], host-parasitoid co-evolution [7] and the evolutionary potential of lineages [8, 9].

However, the molecular changes underlying trait loss have been resolved in only a small number of cases. In a number of these, one or several key genes of the underlying pathway were found to be degraded or missing from the genome. For example, loss of vitamin C production in primates is caused by a frameshift mutation in the gene *Gulo* [10, 11]. Loss of four *opsin* genes, combined with reduced expression of nine important transcriptional factors, underlies eye vestigialization in cave fish [12]. Accumulation of frameshift mutations in enamel-specific genes was found in a number of toothless and enamelless mammal lineages [13, 14]. These studies reveal that loss of a single or a few genes may underlie trait loss. However, the genomic basis of trait loss in highly conserved metabolic traits, where pleiotropic effects may prevent gene loss, is still unresolved.

The expression of a metabolic trait is generally regulated by many interconnected pathways, resulting in a complex network containing intermediate steps encoded by a large number of genes. Trait loss can be the result of a disruption in any of the intermediate steps, but gene network theory predicts that degradation of highly connected genes (so-called hub genes) is usually detrimental or even lethal [15–18] due to pleiotropic functions of these genes. Other studies show that gene pleiotropy also correlates to evolutionary constraints on gene expression [19–21]. Therefore, these genes are unlikely targets for molecular changes underlying decay of complex traits. In contrast, genes with a more peripheral position in the gene network have lower connectivity and fewer pleiotropic functions [22, 23]. When such genes decay or their expression is suppressed, relatively few pleiotropic effects would result on other functions (reviewed in [18]). Regulatory changes might be a common mechanism underlying trait loss, but the role of gene expression and pleiotropic function in relation to trait loss is poorly understood [24].

De novo synthesis of fatty acids is a highly conserved metabolic process involving many deeply conserved and highly pleiotropic genes [25–27]. It is an integral part of the life-history of most animals, enabling them to convert dietary carbohydrates to lipids, which allows storing energy for leaner times and for resource allocation to reproduction. Despite these essential functions of fatty acid synthesis, lack of lipogenesis has repeatedly evolved across the eukaryotic tree of life. For example, several lineages of fungi are fatty acid auxotrophs, caused by loss or degradation of the *fatty acid synthase* gene [28, 29]. Also, multiple insect lineages lack lipogenesis as shown by labelling studies [30–32] or a lack of increase in adult fat reserves, despite feeding on sugar ad libitum [33, 34]. In insects, this recurrent loss of lipogenesis is phylogenetically linked to the parasitoid lifestyle: parasitoid clades of flies, beetles and wasps have lost lipogenesis independently [35]. Parasitoid insects obtain their fatty acids as larvae from their hosts, carrying over a surplus of lipids to the adult stage. Phylogenetic analysis also shows that the ability to synthesize lipids has re-evolved independently in a number of parasitoid wasp species [35]. The repeated regain of lipogenesis, as well as the central role of the lipogenic pathway in carbon metabolism, suggests that the loss of lipogenesis is due to modification of gene expression rather than genetic changes in protein coding regions of the genome.

In this study, we aim to unravel the changes in gene expression underlying the loss of lipogenesis in the parasitoid wasp *Nasonia vitripennis*. In *N. vitripennis*, there is no apparent gene loss or pseudogenization in pathways related to carbohydrate and lipid metabolism [36]. Visser et al. [31] showed that in *N. vitripennis* sugar-feeding does not cause a transcriptional response in several key genes in fatty acid biosynthesis, whereas these are upregulated under the same conditions in *Drosophila melanogaster*. These findings suggest that lipogenesis is decoupled from other metabolic processes in *N. vitripennis*, sharply contrasting with regulation of these processes in other organisms [25, 37, 38].

We characterized the transcriptomic response to sugar-feeding in the non-lipogenic parasitoid wasp *N. vitripennis* and compared it with the transcriptomic response in insects with intact lipogenesis. Since no lipogenic strains of *N. vitripennis* are known, and no other parasitoid species are known that have maintained lipogenic abiliy ([35] shows only species that have regained lipogenesis), we compared *N. vitripennis’* transcriptomic response to that of the well-characterized representative lipogenic species, *D. melanogaster*. Although these species are in different insect orders, the metabolic pathways underlying lipid synthesis are highly conserved among animals, even to the extent that that fruit flies are used as a model for human obesity research (e.g. [39]). An additional advantage is that *D. melanogaster* has a well-annotated genome and it feeds naturally on a sugar-rich diet [37, 40]. We first characterized the transcriptomic response to sugar-feeding for both species separately. Next, we compared these responses between the two species. Our results show that both species respond to sugar-feeding by adjusting transcription of key genes involved in multiple metabolic pathways, but network-based comparative analyses indicate that both organisms evolved contrasting strategies in metabolizing dietary sugar.

## Results

### Global gene expression patterns

We obtained 13.7–20.4 million high-quality 90 bp paired-end reads for each of the 12 libraries (2 species, 2 treatments, 3 biological replicates). 17.7–24.2% of reads were unequivocally mapped to a single locus on the respective reference genomes and kept for subsequent analyses (Additional file 1: Table S1).

**Fig. 1:**
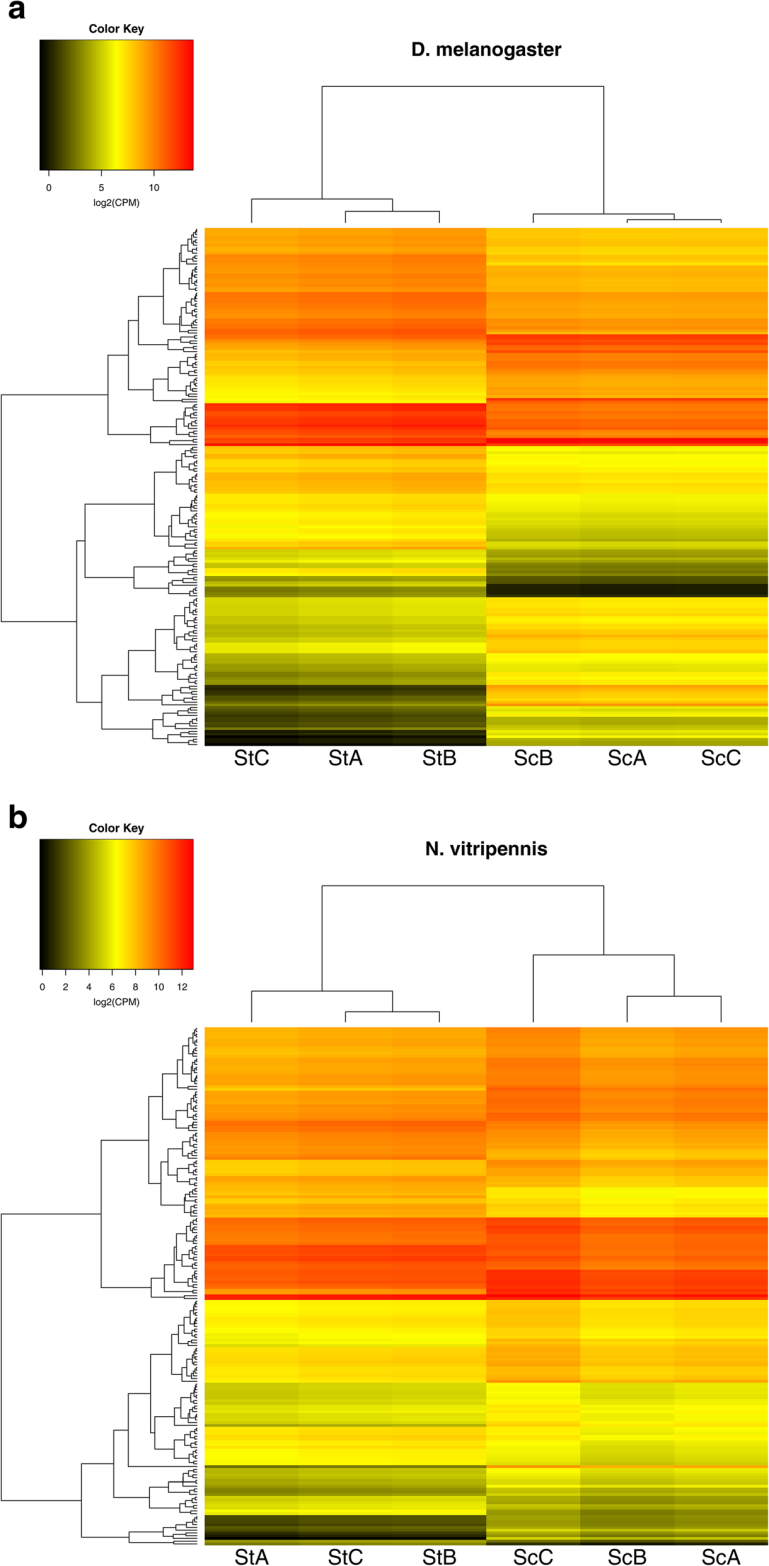
Heatmaps of the *D. melanogaster* (**a**) and *N. vitripennis* (**b**) transcriptomes. CPM = counts per million, St = starved, Sc = sucrose-fed, A/B/C = replicate

We first established that our treatment of 4 hours ad libitum access to sugar had a measurable effect on the insect’s abdominal transcriptome. Heatmaps of these transcriptomes are shown in Fig. 1. The number of enriched gene ontology terms (GO-terms) in the sets of DE genes is presented in Table 1 and Additional file 1: Tables S4 and S5. These significantly enriched GO-terms contain a broad spectrum of processes altered upon sugar-feeding, including terms related to amino acid metabolism, reproduction, and carbohydrate and fat metabolism (Additional file 1: Tables S4 and S5).

**Table 1:** Summary the data sets generated and analysed in this study

Species	Number of genes in reference genome	Number of genes with expression data	Genes up-regulated	Genes down-regulated	GO terms
*D. melanogaster*	17,974	12,977 (72.2%)	84	111	59
*N. vitripennis*	14,321	10,914 (76.2%)	126	62	19

It shows the number of annotated genes for *D. melanogaster* and *N. vitripennis* and the number of genes up- and down-regulated upon sugar feeding with the number of unique Gene Ontology (GO) terms associated to these differentially expressed genes

**Fig. 2:**
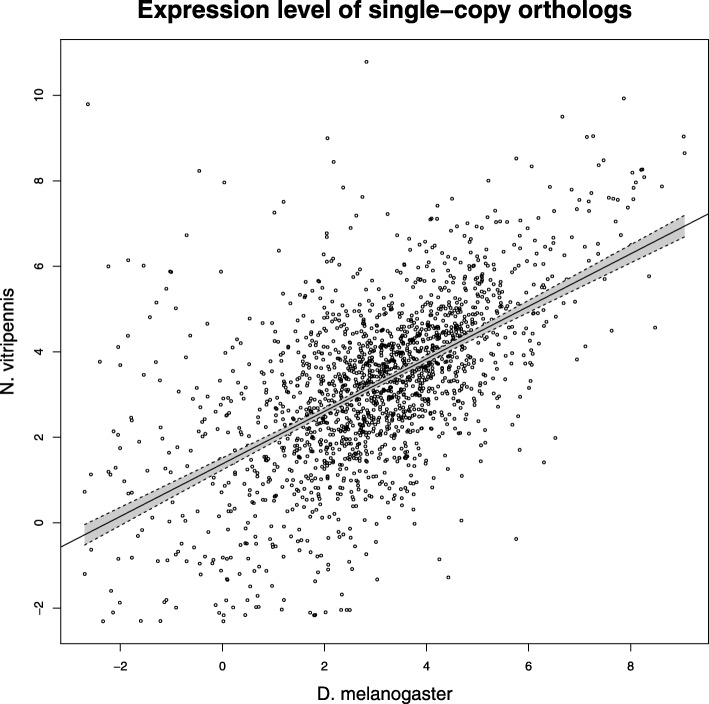
Interspecific correlation of expression level of non-differentially expressed single-copy orthologs between *D. melanogaster* and *N. vitripennis*. Treatments compared were sugar-feeding against brief starvation

To validate a comparison of transcriptomes between different species, we compared the expression level of the 1822 non-differentially expressed single-copy orthologs of *D. melanogaster* against their expression level in *N. vitripennis* (Fig. 2). The overall gene expression patterns are strongly positively correlated between species (Pearson’s r = 0.58, t = 30.155, df = 1820, *p* < 0.0001), despite their phylogenetic distance.

### Transcriptional response to sugar-feeding in *D. melanogaster*

We found significant differences in expression level for 195 genes of *D. melanogaster* after 4 hours ad libitum access to sugar (Additional file 1: Table S2). Fifty-nine GO-terms were enriched in *D. melanogaster* (Fisher’s exact tests, weighted *p*-values < 0.05). Genes related to lipid metabolism were upregulated, including *fatty acid synthase 1* (FBgn0283427) and *lipid storage droplet 1* (FBgn0039114). Genes related to catabolism of amino acids were downregulated upon feeding as well, for instance g*lutamate oxaloacetate transaminase 1* (FBgn0001124).

One thousand one hundred eighty-one genes (11.0% of all genes for which we had expression data) were successfully matched with known reactions in KEGG Pathway. The resulting overview of the active and induced metabolic pathways is presented in Additional file 2: Figure S1A. It shows that the expression of many genes were altered upon sugar-feeding, also in many other pathways other than our focal pathways, e.g. enzymes involved in nucleotide metabolism and amino acid metabolism.

Gene expression is regulated by a range of mechanisms, including non-coding RNAs and cis-regulatory components like transcription factors. Of the differently expressed genes, nine were annotated as non-coding RNAs (Table 2). These were all down-regulated upon sugar-feeding. Only one of the differentially expressed genes was a transcription factor as listed in the transcription factor database REGULATOR: *sugarbabe* (FBgn0033782). It was strongly upregulated in response to sugar-feeding in our experiment.

**Table 2:** Differentially expressed regulatory genes for *D*. *melanogaster* and *N*. *vitripennis* in response to sugar feeding compared to a brief starvation treatment

Species	Type	ID	logFC	logCPM	LR	PValue	Product
*D. melanogaster*	ncRNA^a^	FBgn0262904	−3.98328	1.510955	50.30487	1.42E-09	N/A
		FBgn0263448	−1.89139	1.986294	18.72951	0.002281	N/A
		FBgn0264939	−1.65076	5.328696	20.26412	0.001183	N/A
		FBgn0265150	−2.9169	−1.59583	15.07845	0.011061	N/A
		FBgn0266681	−2.36489	−0.00301	21.90987	0.000639	N/A
		FBgn0266686	−1.39943	3.940312	16.16845	0.007028	N/A
		FBgn0266702	−1.67772	3.741531	20.61357	0.001057	N/A
		FBgn0266705	−1.43091	3.972018	15.94068	0.00746	N/A
		FBgn0267617	−1.36943	4.795973	11.48211	0.047994	N/A
	TF^b^	FBgn0033782	3.196841	2.536466	39.40898	2.35E-07	*sugarbabe*
*N. vitripennis*	ncRNA^a^	LOC103315329	1.22724	5.453971	60.4189	5.23E-12	N/A
		LOC103315513	0.79582	1.559863	13.07675	0.021931	N/A
		LOC103315648	0.884968	7.019906	29.15263	1.55E-05	N/A
		LOC103317395	0.882667	4.463351	17.37046	0.003292	N/A
		LOC103315928	−1.89299	3.81667	122.3051	5.40E-25	N/A
		LOC103315927	−1.53081	4.814787	60.44347	5.23E-12	N/A
	TF^b^	LOC100116547	−1.2319	1.170765	18.09523	0.002415	*muscle segmentation homeobox-like*
		LOC100124032	1.235618	0.241892	13.94669	0.015554	*GATA-binding factor A-like*
		LOC100115031	−0.45739	6.990395	19.5721	0.00129	*Krueppel-like factor 7*
		LOC100122625	0.510109	4.732156	12.6242	0.02631	*uncharacterized LOC100122625*
		LOC100678159	1.169649	3.616958	36.48003	5.91E-07	*Krueppel-like factor 10*
		LOC100115252	0.762056	3.469889	18.70013	0.001836	*forkhead box protein P1*

^a^ncRNA: non-coding RNA

^b^TF: transcription factor

### Transcriptional response to sugar feeding in *N. vitripennis*

We found significant differences in expression level for 188 genes of *N. vitripennis* (Additional file 1: Table S3). Nineteen GO-terms were enriched in *N. vitripennis* (Fisher’s exact tests, weighted *p*-values <0.05). None of the three copies of the gene *fatty acid synthase* (LOC100121447, LOC100122099, LOC100122083) was upregulated in the sugar-fed treatment, but several other genes related to lipid metabolism were: *acetyl-CoA carboxylase* (LOC100123347), *glucose 6-phosphate dehydrogenase* (LOC100120232) and *ATP-citrate lyase* (LOC100119651) and a *citrate transporter* (LOC100118210). A number of genes in other pathways linked to carbohydrate metabolism were differentially regulated upon sugar-feeding: *HMG-CoA synthase 1* (LOC100116401) was upregulated and *phosphoenol pyruvate carboxykinase* (LOC10015526) was downregulated.

**Fig. 3:**
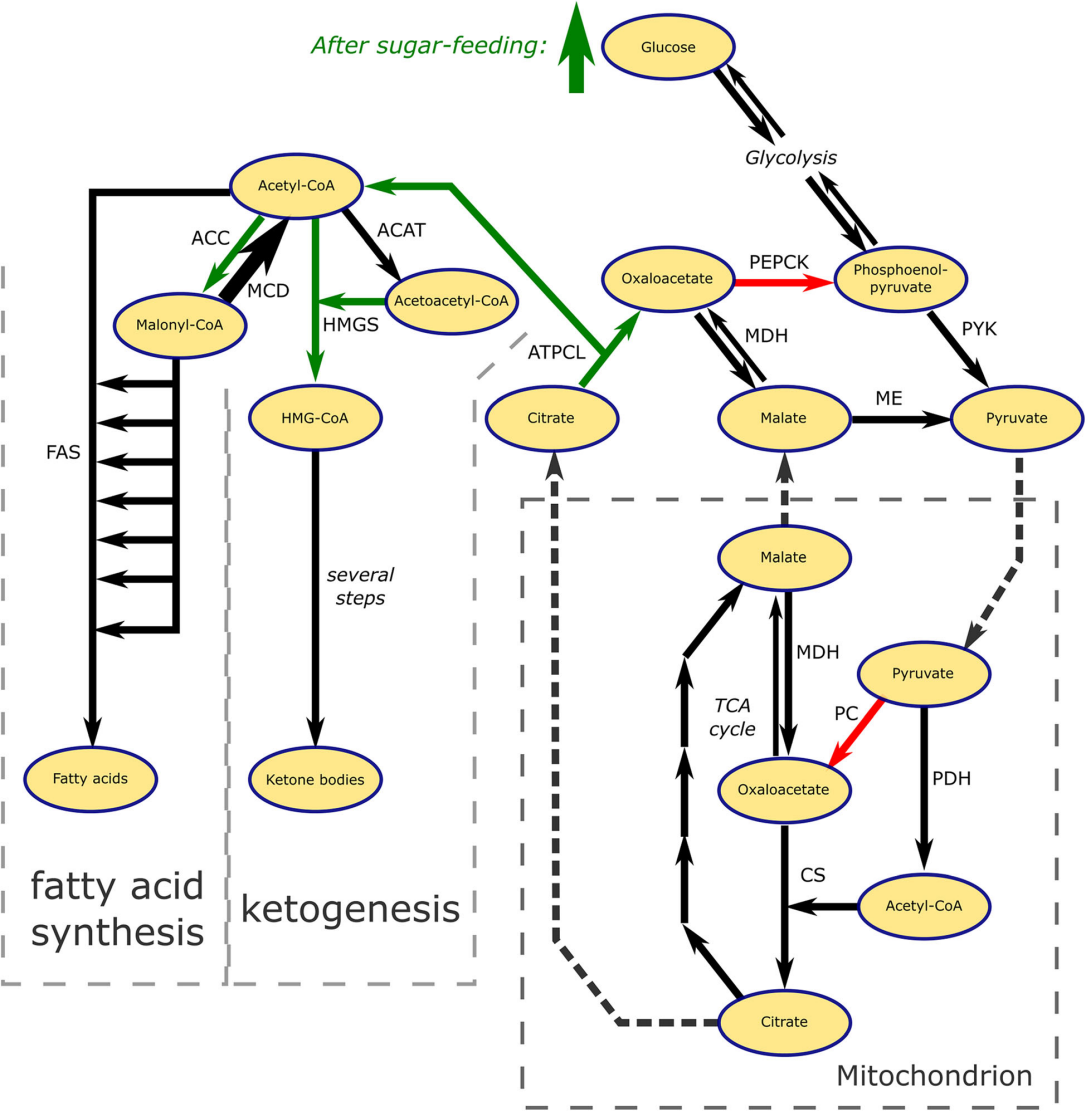
Differential gene expression in acetyl-CoA metabolism of *N. vitripennis* upon sugar feeding. Green and red arrows depict upregulated and downregulated genes, respectively. The thick arrow for MCD symbolize high constitutive expression. Dashed arrows represent transport of metabolites across the mitochondrial membrane

Nine hundred twenty-five genes (8.5% of all genes for which we had expression data) had known reactions in KEGG Pathway. The resulting overview of the active and induced metabolic pathways is presented in Additional file 2: Figure S1B, and the main changes of interest are summarized in Fig. 3.

Of the differently expressed genes in *N. vitripennis*, six genes were annotated as ncRNAs. Four of these were upregulated, two were downregulated (Table 2). Of the transcription factors listed in REGULATOR, four were upregulated and two were downregulated expressed in our experiment (Table 2).

### Comparison between *N. vitripennis* and *D*. *melanogaster*

While the majority of single-copy orthologs showed a strong correlation in expression level between *N. vitripennis* en *D. melanogaster* (see above), there were a number of outliers to this correlation: several orthologs were highly expressed in *N. vitripennis* at all times, but had a much lower expression in *D. melanogaster* (upper-left in Fig. 2). The genes with the most extreme interspecific differences in expression were *radish*, *Phosphodiesterase 1c*, *CG9117*, and *olf413*. *Radish* is a Rap-Like GTPase Activating Protein involved in memory dynamics [41]. *CG9117* is a metallo-beta-lactamase domain-containing protein without further annotation. *olf413* is a copper type II ascorbate-dependent monooxygenase. The opposite expression pattern (highly expressed in *D. melanogaster*, but low in *N. vitripennis*) was found for *Gasp* and *walrus*. *Gasp* is a chitin-binding protein associated with embryonic development. *Walrus* is an electron transfer flavoprotein, probably capable of accepting electrons of several dehydrogenases. The full table of expression levels of non-plastic single-copy orthologs is available in Additional file 1: Table S6.

**Fig. 4:**
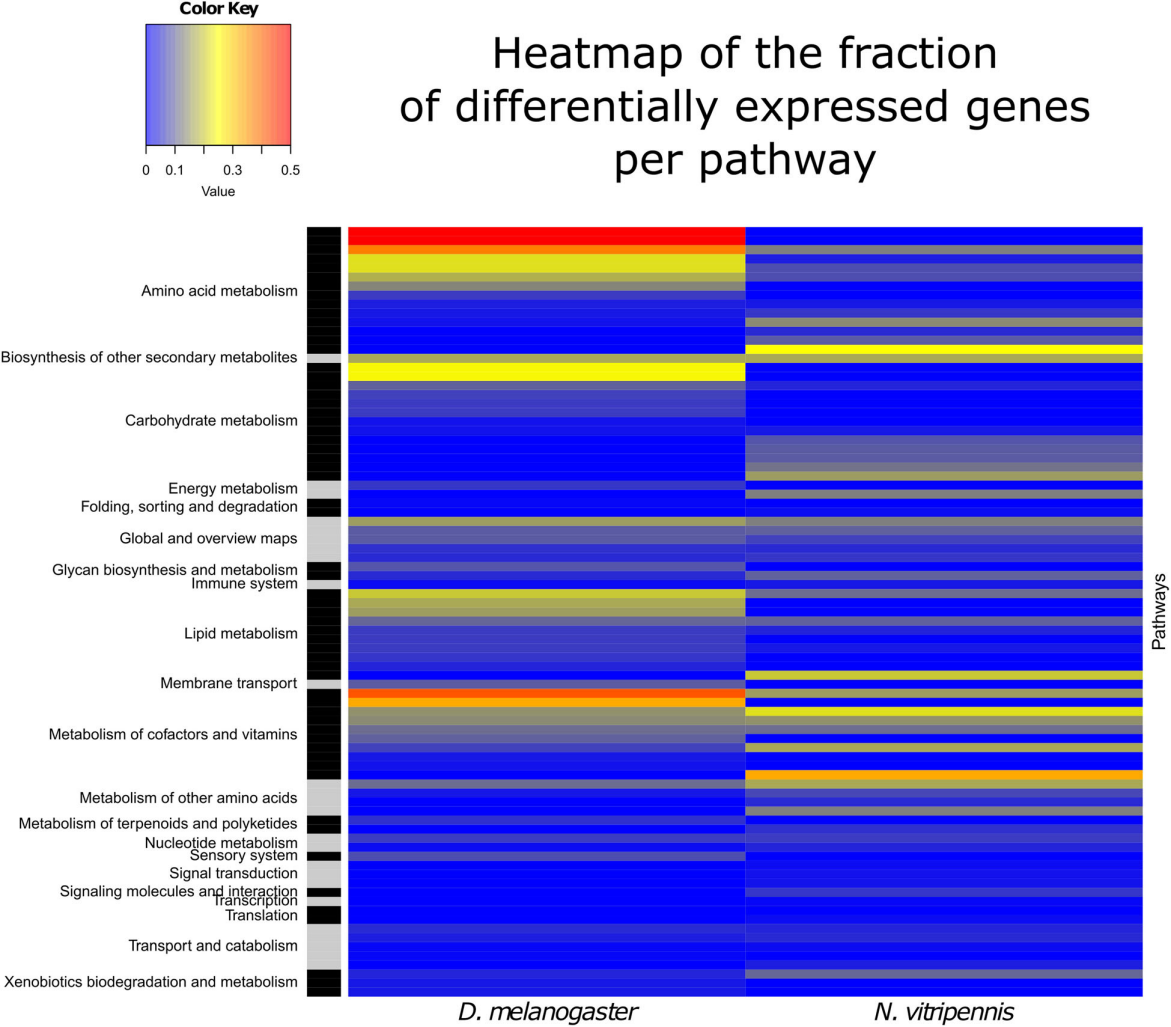
Heatmap of the fraction of genes per KEGG Pathway that was differentially expressed for *D. melanogaster* and *N. vitripennis*. Treatments compared were sucrose feeding against brief starvation. The full table is provided in Additional file 1: Table S7

KEGG-pathways associated with the DE genes were visualised as a heatmap in Fig. 4 for both species. Full tables are available in Additional file 1: Table S7. In the carbohydrate metabolic pathways, *D. melanogaster* showed multiple DE genes in fructose (two genes), galactose (nine genes) and sucrose (eight genes) metabolism, while *N. vitripennis* had no genes differentially expressed in these pathways. By contrast, differentially expressed genes in *N. vitripennis* were involved in the tricarboxylic acid (TCA) cycle (four genes), propanoate and butyrate metabolism (two genes each) and pyruvate metabolism (three genes). There were also divergent responses in the amino acid metabolisms, lipid metabolic pathways and in the pathways of the metabolism of co-factors and vitamins. *Malonyl-CoA decarboxylase* (LOC100120093), the gene that codes for the enzyme that performs the opposite reaction of acetyl-CoA carboxylase, was highly expressed in *N. vitripennis* in both treatments (mean logCPM = 7.415), while this gene was not found in *D. melanogaster*.

### Differential expression correlates to gene pleiotropy

**Fig. 5:**
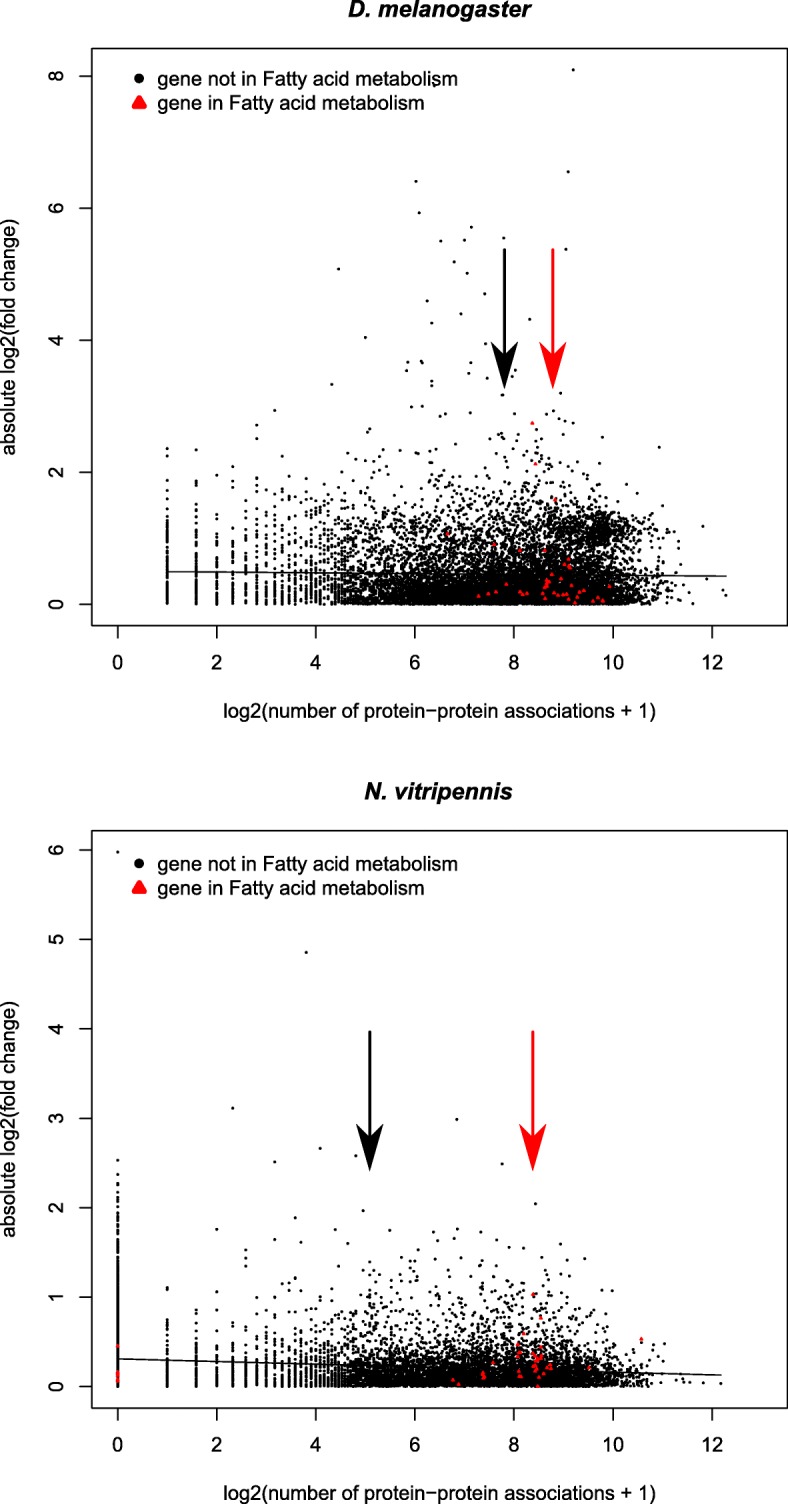
Scatterplots of gene expression upon sugar-feeding against the number of connections for *D. melanogaster* (top) and *N. vitripennis* (bottom). Gene expression is presented as the absolute log2-transformed fold change and connections are the known log2-transformed number of protein-protein associations. There is a significant negative correlation in both species, represented as a linear model in the figures. Red triangles represent genes listed in KEGG Pathway map ‘Fatty acid metabolism’ (map01212), black dots represent all other genes. The median log2-transformed number of protein-protein associations of either gene group is represented by vertical arrows

Figure 5 shows the correlation between absolute fold change of gene expression levels and the number of protein-protein associations (PPA) as a measure for the level of pleiotropy. There was a significant negative correlation between fold change and PPA: Pearson’s r = − 0.187 for *N. vitripennis* (t = − 20.048, df = 11,148, *p* < 0.0001) and r = − 0.025 for *D. melanogaster* (t = − 2.583, df = 11,030, *p* < 0.01). Many genes related to fatty acid metabolism are positioned at the high end of the pleiotropy spectrum: the median number of PPA for genes of *N. vitripennis* from KEGG Pathway map ‘Fatty acid metabolism’ (map01212) is 313, while this number is only 182 for all metabolic pathways combined (map01100).

## Discussion

### Global gene expression patterns

The goal of this study was to characterize the transcriptomic response to sugar-feeding of the non-lipogenic parasitoid wasp *N. vitripennis* in comparison with the lipogenic *D. melanogaster*. The set of differentially expressed genes upon sugar feeding in both species were enriched for a broad spectrum of GO-terms, showing that both species adjust their gene regulation within 4 hours after feeding on a sugar solution. The strong positive correlation of the gene expression levels of conserved (single-copy) genes shows that these patterns are maintained over long evolutionary timeframes (Diptera and Hymenoptera diverged about 340 million years ago [42]) and validates our subsequent comparisons of conserved metabolic pathways between *D. melanogaster* and *N. vitripennis*.

### Transcriptional response to sugar-feeding in *D. melanogaster*

*D. melanogaster* accelerates its fatty acid synthesis after sugar-feeding, as indicated by the upregulation of the key gene *fatty acid synthase 1*. This was expected, as *D. melanogaster* is known to start lipogenesis shortly after sugar-feeding [37]. *Lipid storage droplet 1*, involved in storage of lipids in the fat body, was upregulated concomitantly, as would be expected when lipid production is increased. The downregulation of genes related to catabolism of amino acids upon feeding indicate that the flies may have used amino acids as fuel in the starvation treatment.

Nine non-coding RNAs were down-regulated upon sugar-feeding. The regulatory role of ncRNAs is a dynamic field of research [43–45], without generalized predictions of the function of specific ncRNAs. We show here that these nine ncRNAs are either co-regulated with the metabolic genes of *D. melanogaster* or regulating them. Note that our library preparation methods excluded all miRNAs. The transcription factor *sugarbabe* was strongly upregulated in our experiment. In an earlier microarray study this transcription factor was shown to be upregulated in *D. melanogaster* larvae shortly after sugar-feeding [37].

### Transcriptional response to sugar-feeding in *N. vitripennis*

Fatty acid synthesis was not activated in response to sugar-feeding in *N. vitripennis* as indicated by the lack of a response of the three *fatty acid synthases* in the sugar-fed treatment. This is consistent with the reported lack of lipogenesis in *N. vitripennis* after sugar-feeding [31, 46], as no fatty acid synthesis is possible without the key enzyme encoded by *fatty acid synthase*. However, several other genes that play a direct or indirect role in the fatty acid synthesis pathway were upregulated. The enzyme Acetyl-CoA Carboxylase (ACC) adds a carboxyl group to acetyl-CoA yielding malonyl-CoA which is required for fatty acid synthesis. High levels of malonyl-CoA also inhibit the activity of Carnitine Acyltransferase I (CAT1), preventing fatty acid transport to the mitochondrion, thereby limiting β-oxidation. Glucose-6-phosphate Dehydrogenase (G6PDH) produces NADPH^+^, required for anabolic processes like fatty acid synthesis. Activity of ATP-Citrate Lyase (ATP-CL) is indicative of acetyl-CoA transport from the mitochondrion to the cytoplasm, generally implicated in fatty acid synthesis. The upregulated *citrate transporter* potentially facilitates this transport. Citrate in the cytoplasm stimulates the activity of ACC.

These changes in gene expression in response to sugar-feeding indicate that most of the gene expression patterns in the lipid metabolic pathway are preserved with the exception of the key step involving upregulation of *fatty acid synthase*. In other cases of trait loss, changes in the underlying pathway have been found to extend to multiple genes, e.g. in the case of loss of vision [12]. Evolutionary theory predicts that degradation of a key gene in a pathway will be followed by mutation accumulation in other genes in the pathway, because they are no longer under selection as soon as the phenotypic function is lost. This is in contrast with what is observed in the lipid synthesis pathway of *N. vitripennis*: even though there is a phenotypic lack of lipogenesis, we only found regulatory changes in *fatty acid synthase*, not in other genes of the lipid synthesis pathway. A possible explanation for this paradox could be evolutionary constraints on these genes due to pleiotropy. If the enzymes encoded by genes in the lipid synthesis pathway are also active in other metabolic processes, regulatory changes may be prevented resulting in decoupling of the different processes.

A number of other pathways linked to carbohydrate metabolism were differentially regulated upon sugar-feeding. For example, gluconeogenesis was decelerated as indicated by a downregulation of *phosphoenol pyruvate carboxykinase* (*pepck*) transcripts. A reduction of gluconeogenesis is expected to be concomitant with a reduction in ketogenesis. However, the upregulation of *hydroxymethylglutaryl-CoA synthase 1* (*hmgs1*) indicates that ketogenesis was accelerated. Catabolism of acetyl-CoA through increased ketogenesis might be linked to loss of lipogenesis in *N. vitripennis*. An earlier study using qPCR to assess gene transcriptional responses to sugar-feeding in *N. vitripennis*, found congruent results for key genes involved in carbohydrate, fatty acid, and glycerolipid metabolism, including *acetyl-CoA carboxylase*, *ATP citrate lyase*, *glucose-6-phosphate dehydrogenase* and *phosphoenolpyruvate carboxykinase* [31].

Four non-coding RNAs were upregulated and two were downregulated in *N. vitripennis*. This suggests that these non-coding RNAs in *N. vitripennis* could be involved in regulating its diverging metabolic response to sugar-feeding. Four transcription factors were upregulated and two were downregulated in our experiment. As with the non-coding RNAs, we lack information on their targets and mechanism. It is possible that one of these, or their combined effects, regulate the decoupling of lipogenesis from sugar metabolism.

### Comparison between *N. vitripennis* and *D. melanogaster*

It is unclear how the outliers in the comparison of expression levels of single-copy orthologs relate to differences in lipogenic abilities between the studied species. Similarly, it is unknown what the differences in expression patterns of non-coding RNAs and transcription factors mean. Nonetheless, our data indicate that *D. melanogaster* accelerates fatty acid synthesis upon sugar-feeding, while some key components of this pathway in *N. vitripennis* lack a response. In the carbohydrate metabolic pathways, *D. melanogaster* showed many differentially expressed genes in fructose, galactose and sucrose metabolism, while *N. vitripennis* had no genes differentially expressed in these pathways. By contrast, differentially expressed genes in *N. vitripennis* were involved in the TCA cycle, propanoate and butyrate metabolism, and pyruvate metabolism. Moreover, there are contrasting responses in the amino acid metabolisms, lipid metabolic pathways and in the pathways of the metabolism of co-factors and vitamins. Although we currently cannot exclude temporal differences in the patterns of gene expression, these results suggest that these species use a different method of metabolizing dietary sugar and have a divergent response to sugar-feeding overall.

### Differential expression correlates to gene pleiotropy

The negative correlation between fold change and the number of connections (PPA) indicates that highly pleiotropic genes are constrained in their extent of up- or downregulation, which is particularly relevant when the insect’s metabolism is under selection. Many genes related to fatty acid metabolism are positioned at the high end of the pleiotropy spectrum, which means that most genes are rather constrained in their change in gene expression. We expected that the regulatory reorganization of fatty acid and acetyl-CoA metabolism of *N. vitripennis* is likely to be directed by genes of low pleiotropy. A good candidate with few known PPA would be *malonyl-CoA decarboxylase* (LOC100120093). This gene encodes the enzyme that converts one of the substrates for Fatty Acid Synthase, malonyl-CoA, to acetyl-CoA. It has a high constitutive expression in *N. vitripennis* in both feeding treatments. The high expression of this gene could therefore potentially deplete available malonyl-CoA, which would impede fatty acid synthesis. *Malonyl-CoA decarboxylase* has been lost in *D. melanogaster*. Other potential candidates having low PPA that likely underlie loss of lipogenesis are genes coding for enzymes catabolizing acetyl-CoA, or otherwise disposing of it, such as genes involved in ketogenesis and the TCA cycle. Indeed, we show *HMG-CoA synthase 1*, an intermediate step in ketogenesis, to be upregulated in *N. vitripennis* upon sugar-feeding.

## Conclusion

We characterized the gene expression patterns of the non-lipogenic *N. vitripennis* upon sugar-feeding in order to find clues to the molecular mechanism underlying the evolutionary loss of lipogenesis in this species. Animals feeding on sugar generally upregulate their lipogenic pathways [25, 47], as we observed for the lipogenic *D. melanogaster*. *N. vitripennis* seems to have evolved a regulatory mechanism that decouples sugar ingestion and lipogenesis. Rather than storing dietary sugar in the form of fat, it uses the high expression of *malonyl-CoA decarboxylase* to counteract the activity of Acetyl-CoA Carboxylase and subsequently direct the acetyl-CoA to ketogenesis. The carbohydrates from sugar-feeding are used in somatic maintenance and as a source of energy for physical activity as parasitoid wasps feeding on sugar live longer and loose less fat reserves ([48] and references therein). Catabolizing excess carbohydrates via ketone bodies probably helps to avoid adverse effects of a high glucose diet and simultaneously enable retention of fat stores carried over from the larval stage.

Our results raise the question why these lipogenesis-genes are maintained and expressed at detectable levels in *N. vitripennis* despite apparent lack of function. One explanation could be that the genes involved in lipogenesis have undetected subtle forms of gene degradation that impair enzyme function. Alternatively, genes involved in lipogenesis could be under purifying selection through their pleiotropic effects. The regulatory mechanism hypothesized here could be a way of blocking lipogenesis in adult wasps, while maintaining the genes’ other pleiotropic functions. This could be tested in a knockdown experiment, for example by using RNAi. Future studies on the regulatory network of lipogenesis would give mechanistic insights in these evolutionary constraints.

## Methods

### Study species

We used *Drosophila melanogaster* (Diptera: Drosophilidae) Bloomington stock 2057 (RRID:BDSC_2057) [49] and *Nasonia vitripennis* (Hymenoptera: Pteromalidae) strain AsymCX [36] in our experiments since the reference genomes originated from these strains. Both species naturally feed on sugary solutions that are lipid-free, e.g. nectar and honeydew. All strains were kept at 25 °C, 75% relative humidity and 16:8 h L:D cycle prior to and throughout the experiment.

### Experimental setup

All newly emerged insects were kept without access to food for 24-36 h (only water). This pre-treatment period ensured that all were hungry and eager to feed. Next, mated females were randomly assigned to either of two treatments: starved (St) and sucrose-fed (Sc). Starved insects were kept without food for another 4 h; sucrose-fed insects were given ad libitum access to a sucrose solution (20% *w*/*v*). This is a sugar concentration similar to natural sources of carbohydrates encountered by free-living insects such as nectar [50] and honeydew [51, 52]. All insects of the latter treatment were observed to feed within a few minutes. After exactly 4 h all insects where killed by freezing in liquid nitrogen.

### Tissue collection and RNA extraction

Each experimental condition consisted of 10 individual females and the experiment was repeated three times, yielding three independent biological replicates. Frozen females were dissected on a clean liquid nitrogen-cooled steel block: only abdomens were retained for total RNA extraction. For *N. vitripennis*, eight females were pooled per RNA extraction. For *D. melanogaster*, each sample of RNA was extracted from two pools of four females which were subsequently combined. The remaining two individuals per replicate were stored frozen as backup. RNA was extracted using the Promega SV Total RNA Isolation System kit (Promega Corporation, USA) following manufacturer’s instructions except that RNA extracts were eluted in 30 μL water. RNA concentrations were measured on a NanoDrop 2000 (Thermo Scientific) and the RNA Integrity Number was measured on a BioAnalyzer 2100 (Agilent). All samples had sufficient quantities of the required quality of RNA (Additional file 1: Table S1).

### Illumina sequencing

All samples were sent to the Beijing Genomics Institute where library preparation and sequencing were performed. This strand-specific TruSeq library preparation included poly-A RNA purification, mRNA fragmentation, cDNA synthesis from size-selected fragments using random primers and adapter ligation for sample identification. Resulting short-insert libraries were pooled and sequenced (90 bp paired-end) on one lane of Illumina HiSeq2000. This yielded 13–20 M reads per sample (detailed in Additional file 1: Table S1). Raw sequence data have been deposited in the NCBI Short Read Archive under the study accession number SRP127311.

### Transcriptomes and reference genomes

Sequence reads were checked for quality using FastQC version 0.10.1 [53]: only high quality clean reads were delivered. The reference genome annotations used for *N. vitripennis* and *D. melanogaster* were GCF000002325.3_Nvit_2.1 [54] and dmel_r6.11 [55], respectively.

Indices to the reference genomes were built with Bowtie2-build version 2.2.4 [56]. Reads were mapped to the respective reference genomes using TopHat2 version 2.0.13 [57] with the following settings: -r 0 -p 4 --library-type fr-firststrand. Statistics of mapping success are reported in Additional file 1: Table S1. Alignment files were converted to SAM-format using samtools version 0.1.19 [58]. GFF-files were converted to comply to HTSeq format requirements with a python script. Expression levels were counted per gene using HTSeq version 0.6.1 [59] with default settings. Only genes for which five or more libraries (for each species separately) had non-zero counts were retained because zero counts could cause spurious similarity between samples [60].

Differential gene expression analyses were performed in EdgeR version 3.10.2 [61] as recommended by Guo et al. [62]. Gene expression was compared between the two treatments (starved vs. sucrose-fed) for each species separately. Normalization factors were calculated with the function calcNormFactors with default settings. A negative generalized log-linear model was fitted to the data and a Likelihood ratio test was used to obtain *P*-values [63]. P-values were corrected for multiple testing using the R function p.adjust following the method of Benjamini-Hochberg [64]. A gene was considered significantly differentially expressed when the *p*-value associated with this comparison was below 0.05 after FDR correction.

### Gene ontology enrichment analyses

For each species, the set of differentially expressed genes was checked for enriched GO-terms with the R-package ‘topGO’ version 2.20.0 [65]. We used the weighed P-values that TopGO calculates using an algorithm that weighs statistical significances of higher GO categories by the significance of its lower categories: Higher categories are only recovered as significant when more genes are found at that category than expected by chance. GO-indexes for the *D. melanogaster* gene annotation were downloaded from FlyBase [55]. For *N. vitripennis* we used the Blast2GO-based GO-index for the Nvit2.1 assembly [66]. Only GO-terms in the category ‘biological process’ were considered.

### Orthologous gene-based comparisons

Orthologs of *D. melanogaster* and *N. vitripennis* were obtained from OrthoDB7 [67]. This list of orthologous groups is based on previous versions of the *D. melanogaster* and *N. vitripennis* genome annotations and gene predictions that are not supported in the current annotations were omitted from the analysis. All single-copy orthologs were extracted from the database. Next, we calculated the Pearson’s correlation of the expression levels of the non-differentially expressed orthologs of *N. vitripennis* and *D. melanogaster*.

### Species-specific transcriptional responses

For each species, we manually checked the lists of differentially expressed genes for involvement in lipogenesis, regulation of lipogenesis, or carbohydrate metabolism pathways. Sets of differentially expressed genes between treatments were screened for regulatory components: (1) transcription factors, as listed in REGULATOR [68] and (2) non-coding RNAs, as annotated in the respective reference genomes.

Gene pleiotropy was estimated by the number of protein-protein assocations (PPA) as collected in the database STRINGdb ([69]. The absolute change in expression level (logFC) in our treatments was tested for a correlation with the number of connections (PPA) after log2 transformation.

### Visualization of KEGG-pathways

Each gene for which we obtained sufficient expression data was queried against the KEGG Pathway database [70, 71] in order to match it to a specific enzymatic reaction number. This analysis was repeated for *D. melanogaster* and *N. vitripennis* independently. These reactions were coupled to the corresponding gene expression data in our transcriptomes and imported into iPath2 [26, 72] to visualize the metabolic maps of both species. Our functional interpretation of biochemical pathways is based on Berg et al. [73].

### Pathway-based comparison between transcriptomes of *D. melanogaster* and *N. vitripennis*

The number of DE genes per KEGG Pathway were summed for both species and divided by the total number of genes that each species had for that pathway. This fraction of induced genes per pathway is presented as a heatmap using the R function heatmap.2 from package gplots.

## Additional files

Additional file 1: **Table S1.** Sample quality measurements, number of recovered reads per sample and mapping success. **Table S2.** Differentially expressed genes of *D. melanogaster*. **Table S3.** Differentially expressed genes of *N. vitripennis*. **Table S4.** Enriched GO-terms of the differentially expressed genes of *D. melanogaster*. **Table S5.** Enriched GO-terms of the differentially expressed genes of *N. vitripennis*. **Table S6.** Non-plastic genes sorted by residual differences in expression level. **Table S7.** KEGG-pathways associated with the differentially expressed genes per species. (XLS 6305 kb)

Additional file 2: Overview of the active and altered metabolic pathways in abdomens of **Figure S1A.** *D. melanogaster*, and **Figure S1B.** *N. vitripennis.* Green lines represent accelerated reactions upon sugar-feeding as inferred from upregulation of the underlying gene. Red lines represent decelerated reactions. Line thickness is linearly scaled to the expression level (logFC). (PNG 9421 kb)

## Abbreviations

DE: differentially expressed
GO: gene ontology
logFC: base 2 logarithm of fold change
PPA: protein-protein associations
TCA: tricarboxylic acid
