# Supplementary figures and images for: Gene expression changes associated with the evolutionary loss of a metabolic trait: lack of lipogenesis in parasitoids
Source: BMC Genomics. 2019 Apr 23;20:309. doi: 10.1186/s12864-019-5673-6 (PMC6480896; doi:10.1186/s12864-019-5673-6)

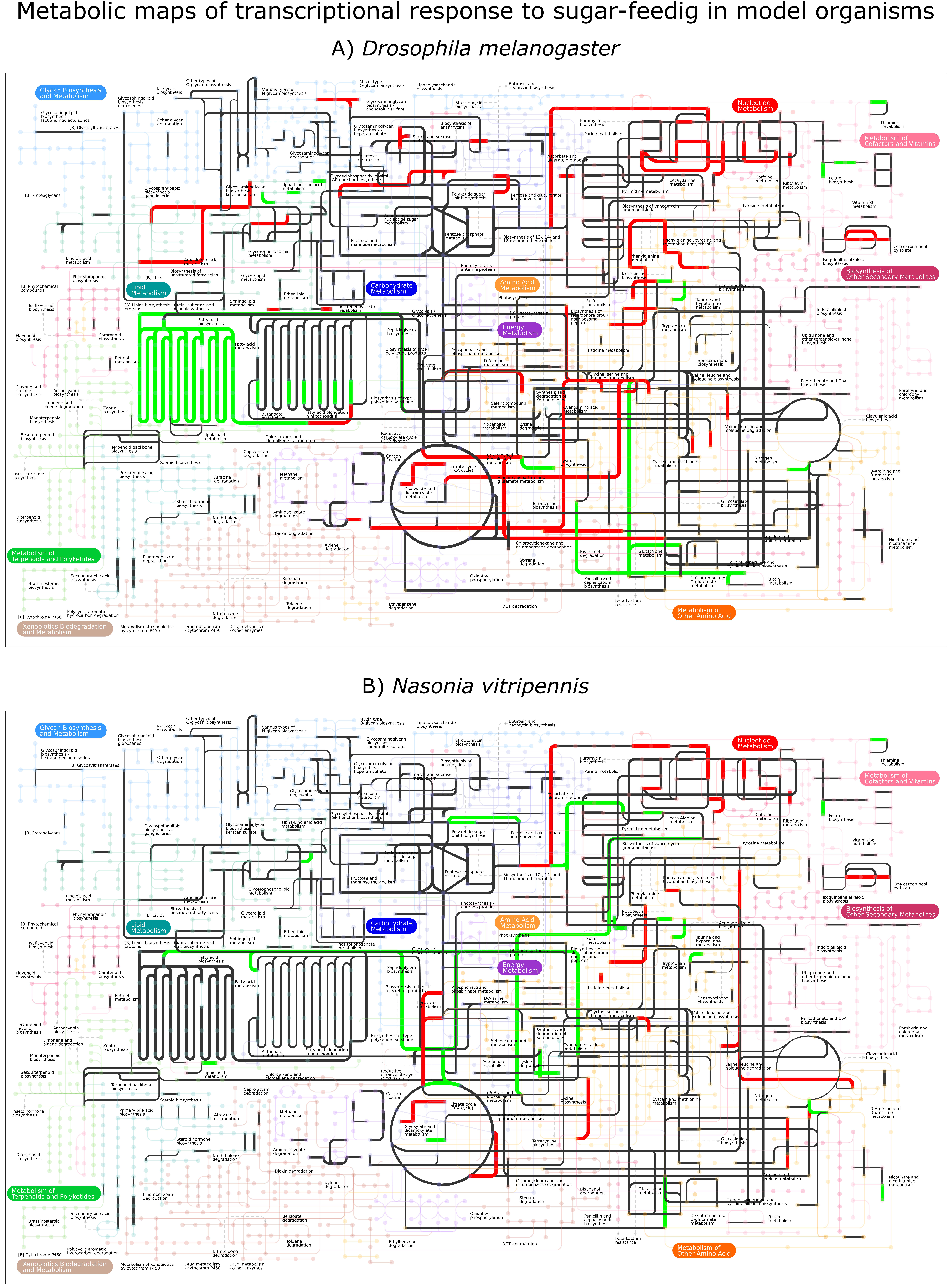

Supplement: Supplementary file 2 — Overview of the active and altered metabolic pathways in abdomens of Figure S1A. D. melanogaster, and Figure S1B. N. vitripennis. Green lines represent accelerated reactions upon sugar-feeding as inferred from upregulation of the underlying gene. Red lines represent decelerated reactions. Line thickness is linearly scaled to the expression level (logFC). (PNG 9421 kb) [file 12864_2019_5673_MOESM2_ESM.png]
